# Prevalence and recurrence rates of spontaneous pneumothorax in patients with diffuse cystic lung diseases in China

**DOI:** 10.1186/s13023-025-03587-6

**Published:** 2025-02-11

**Authors:** Rui Wang, Xianmeng Chen, Shicheng Xu, Xianliang Jiang, Jinli Liu, Xuehan Liu, Jay H. Ryu, Xiaowen Hu

**Affiliations:** 1 Department of Pulmonary and Critical Care Medicine, the First Affiliated Hospital of USTC, University of Science and Technology of China, Hefei, China; 2 WanNan Medical College, Wuhu, China; 3 Center for Diagnosis and Management of Rare Diseases, the First Affiliated Hospital of USTC, University of Science and Technology of China, Hefei, China; 4 Department of Radiology, the First Affiliated Hospital of USTC, University of Science and Technology of China, Hefei, China; 5 Department of Thoracic Surgery, the First Affiliated Hospital of USTC, University of Science and Technology of China, Hefei, China; 6 Department of Dermatology, the First Affiliated Hospital of USTC, University of Science and Technology of China, Hefei, China; 7 Office of Scientific Research, Division of Life Sciences and Medicine, the First Affiliated Hospital of USTC, University of Science and Technology of China, Hefei, China; 8 Division of Pulmonary and Critical Care Medicine, Mayo Clinic, Rochester, MN USA

**Keywords:** Diffuse cystic lung diseases, Lymphangioleiomyomatosis, Pulmonary langerhans cell histiocytosis, Birt-Hogg-Dubé syndrome, Pneumothorax, Sjögren’s syndrome

## Abstract

**Objectives:**

To investigate the prevalence and recurrence rates of spontaneous pneumothorax (SP) in patients with diffuse cystic lung diseases (DCLDs).

**Methods:**

We retrospectively identified and analyzed medical records of patients with DCLDs encountered at the First Affiliated Hospital of University of Science and Technology of China from Jan 1, 2017 to December 31, 2023.

**Results:**

A total of 289 patients were identified with DCLDs; 212 females and 77 males, with a median age of 48 years (range, 18–81 years). Among them, 89 (31%) patients had experienced SP; 59% among 115 patients with Birt-Hogg-Dubé (BHD), 34% of 41 patients with lymphangioleiomyomatosis (LAM, all women), 36% of 11 patients with pulmonary Langerhans cell histiocytosis (PLCH), none of 57 patients with Sjögren’s syndrome-associated diffuse cystic lung disease (SS-DCLD), and 5% of 65 patients with no identifiable underlying disease (χ² = 90.585, *P* < 0.001). The overall recurrence rate of SP was higher with observation or chest tube placement strategy compared to surgical intervention, 59% vs. 11% (*P <* 0.001, 95% CI [0.1, -0.4]), respectively. The recurrence rate after surgical management was significantly lower compared to conservative management in patients with BHD (10% vs. 69%, *P <* 0.001, 95% CI [0.1, 0.3]) and LAM (20% vs. 57%, *P* = 0.322, 95% CI [0.1, 2.1]). Among patients with BHD, LAM, and PLCH, those who had pneumothorax as the initial presentation were diagnosed of their underlying disease at a significantly younger age (42.2 ± 13.0 years) compared to those without pneumothorax (48.1 ± 11.8 years) (*P* = 0.032, 95% CI [-8.24, -0.36]). Notably, eight of LAM patients who were treated with sirolimus after the initial SP did not experience recurrence of SP.

**Conclusion:**

The risk of SP secondary to DCLDs was highest in patients with BHD, followed by those with PLCH and LAM. It was extremely low in SS-DCLD. Pneumothorax as the initial presentation often facilitated diagnosis of the underlying disease. Surgical treatment was associated with a lower recurrence rate of SP compared to nonsurgical management. In addition, sirolimus therapy may reduce the risk of pneumothorax recurrence in patients with LAM.

**Supplementary Information:**

The online version contains supplementary material available at 10.1186/s13023-025-03587-6.

## Introduction

Primary spontaneous pneumothorax (no underlying lung disease) occurs with an annual incidence rates ranging from 1.2 to 9.8 per 100,000 women and 7.4 to 24 per 100,000 men [1, 2]. A recent study from France demonstrated an estimated annual rate of 22.7 cases per 100,000 population [3]. Pneumothorax is associated with significant morbidity and economic burdens on society [4]. In some patients with spontaneous pneumothorax (SP), underlying lung disease can be identified (i.e., secondary spontaneous pneumothorax), among which are diffuse cystic lung diseases (DCLDs) [5].

DCLDs represent a spectrum of heterogeneous diseases that manifest multiple cystic lesions in the lung on computed tomography (CT) of the chest [6]. Common causes of DCLDs include Birt-Hogg-Dubé syndrome (BHD), lymphangioleiomyomatosis (LAM), pulmonary Langerhans cell histiocytosis (PLCH), and Sjögren’s syndrome (SS) [7]. Despite the varying pathophysiological mechanisms among these DCLDs, the presence of cysts in the lung parenchyma increases the risk of SP. In BHD, SP has been reported to affect up to 76% of patients [8]. For those with LAM, about 57% will develop SP [9]. In PLCH, the incidence is around 15–20% [10]. In contrast, SP has rarely been reported in patients with SS-associated diffuse cystic lung disease (SS-DCLD). Although several case series have documented pneumothorax in patients with SS [11–13], the risk of recurrence has not been well-defined. In addition, there have been conflicting data regarding the recurrence rate of pneumothorax associated with surgical treatment compared to nonsurgical management in those with DCLDs [14–16].

To date, no single center has systematically surveyed a large cohort to analyze the clinical characteristics and recurrence rates for patients with pneumothorax associated with DCLDs, and no cohort study has examined the risk of pneumothorax in patients with SS-DCLD. We conducted this study to investigate the demographic and clinical characteristics, prevalence of pneumothorax, management, and recurrence rates among patients with DCLDs encountered at a Chinese tertiary-referral medical center.

## Materials and methods

### Patient selection

We identified all patients diagnosed with DCLDs at the First Affiliated Hospital of the University of Science and Technology of China (USTC) over a 7-year period from January 1, 2017, to December 31, 2023. Diffuse cystic lung disease (DCLD) was defined as the presence of 3 or more air-filled spaces with thin walls (less than 2 mm), and clearly demarcated from normal lung tissue as depicted on CT or high-resolution CT (HRCT) scan of the chest. We excluded patients under 18 years of age.

**Fig. 1:**
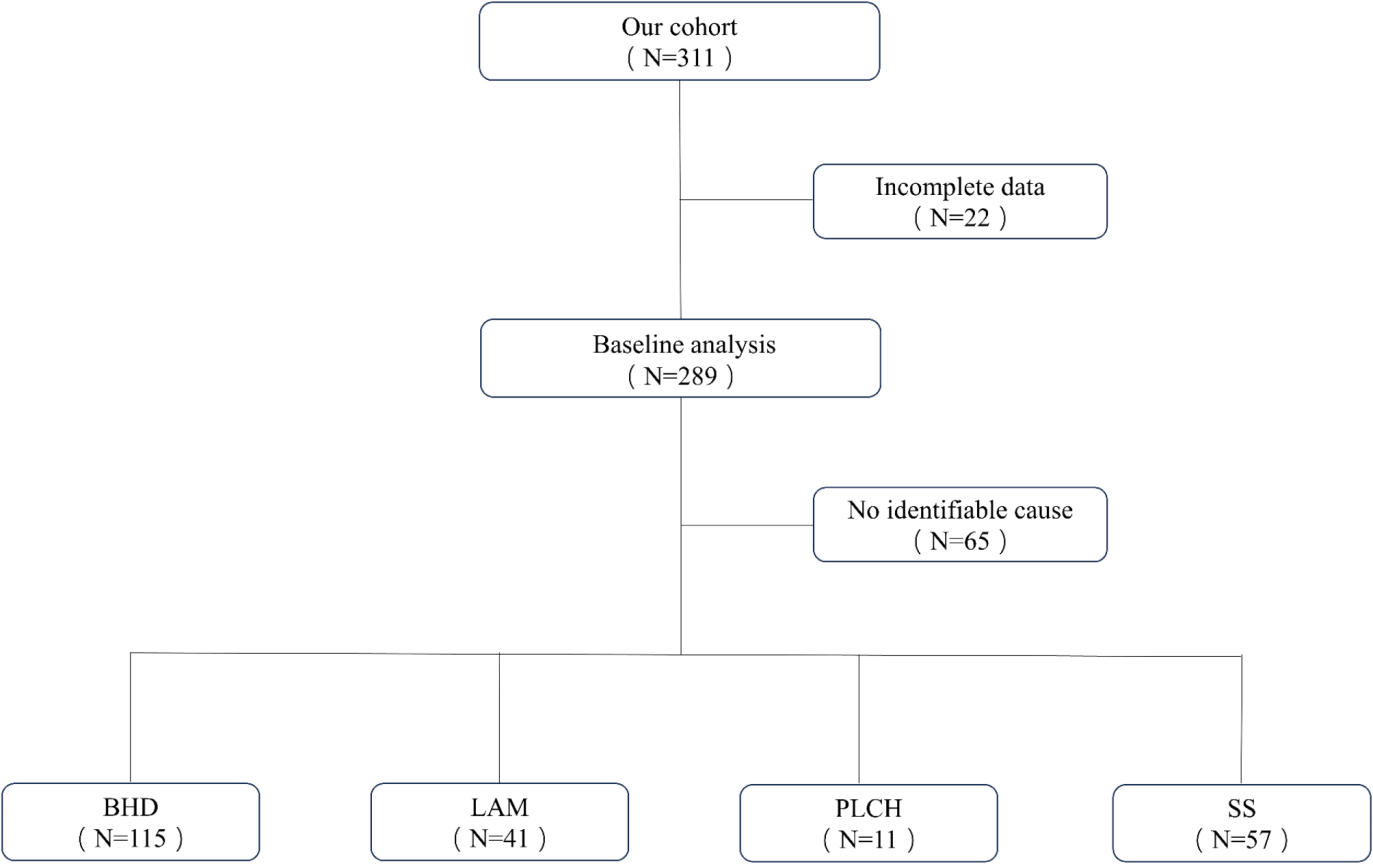
Study flow chart. BHD = Birt-Hogg-Dubé Syndrome; LAM = lymphangioleiomyomatosis; PLCH = pulmonary Langerhans cell histiocytosis; SS = Sjögren’s syndrome

Of 311 patients with DCLDs identified. We excluded 22 patients with incomplete clinical data; a total of 289 patients were included in this study and included 115 with BHD, 41 with LAM, 57 with SS, 11 with PLCH, and 65 with no identifiable underlying cause for DCLDs (Fig. 1). The study was approved by the Ethical Committee of the First Affiliated Hospital of the USTC in Anhui Province (No. 2024-RE-117). Clinical data extracted included gender, age at diagnosis; the number, date and side of pneumothorax, along with recurrences of SP.

### Diagnostic approach

The stepwise diagnostic procedure for diagnostic evaluation of DCLDs followed the comprehensive framework proposed by Cui et al. [7], with the diagnostic approach for BHD further refined based on the detailed methodology developed by Zhang et al. [17] from our medical center.

### Diagnostic criteria

BHD was diagnosed according to the criteria proposed by the European BHD consortium [18], LAM was diagnosed according to the criteria of the 2017 guidelines from the American Thoracic Society and Japanese Respiratory Society (ATS/JRS) [9]. The diagnosis of SS [19] and PLCH [20] was made based on published criteria. When pulmonary manifestations were consistent with LCH, histopathologic confirmation of diagnosis included biopsy of an extrathoracic lesion, such as bone [10]. All SS-DCLD patients met the characteristic pulmonary imaging features of SS [21].

### Follow-up

All patients were followed up until February 1, 2024. Pneumothorax data from the initial presentation of DCLDs to the last follow-up were gathered through medical record review and telephone inquiries. The recurrence of a pneumothorax was defined as one that occurred on the ipsilateral side > 7 days after a pneumothorax had resolved. The diagnosis of pneumothorax was verified on chest imaging. Data on the date, location, frequency, and treatment of pneumothorax were collected and confirmed.

### Statistical analysis

Continuous variables were expressed as mean and standard deviation, and compared by independent sample t-test. Categorical variables were described as frequencies and percentages, and compared by the chi-square test and Fisher’s exact test. A value of *p* < 0.05 was considered statistically significant. All data were analyzed by SPSS version 26.0.

## Results

A total of 289 patients with diffuse cystic changes on chest CT/HRCT at the First Affiliated Hospital of USTC between January 2017 and December 2023 were enrolled. Of these, 221 patients were from our Rare Lung Disease Clinic and 68 were hospitalized patients at presentation. The majority of the study cohort were female (212/289, 73%). All patients with DCLDs were of Han ethnic group. The median age of patients at the time of diagnosis was 48 years (range, 18–81 years). Only 12% (36/289) patients had a smoking history. Overall, 31% (89/289) of those with DCLDs had experienced at least one episode of pneumothorax, 18% (52/289) had endured recurrent episodes of pneumothorax. A total of 208 pneumothorax episodes occurred, with 75 (36%) treated surgically and 133 managed conservatively. Among 89 pneumothorax patients, 43 (48%) were diagnosed with DCLDs during their first episode; 33 (77%) underwent surgery, and 10 received conservative treatment. The remaining 46 patients were initially managed conservatively.

Among the surgeries, there were 38 bullectomies alone, 27 bullectomies with pleurodesis (67% mechanical, 33% chemical), 1 lung transplant, and 9 bullectomies with unclear information about pleurodesis. Eight recurrences were reported: 5 after bullectomy alone and 3 after bullectomy with pleurodesis. All surgeries, except for one lung transplant, were thoracoscopic (80% single-port, 20% multi-port). No pleurectomies were performed. Lung transplantation was performed on a patient with LAM; there were no other lung transplants in our study cohort. Identifiable causes underlying DCLDs included BHD, LAM, PLCH, and SS, accounting for 78% of those with DCLDs. There were 22% of patients with DCLDs who had no identifiable underlying cause (Table 1). The overall recurrence rate of SP was significantly higher with observation or chest tube placement compared to surgical intervention, 59% vs. 11%, (*P* < 0.001, 95% CI [0.1, 0.4]), respectively (Table 2).

**Table 1:** Clinical characteristics of patients with DCLDs

	BHD			LAM			PLCH			SS			No identifiable cause		
Variable	PTX (*n* = 47)	No-PTX	*P* value (*n* = 68)	PTX (*n* = 27)	No-PTX	*P* value (*n* = 14)	PTX (*n* = 4)	No-PTX	*P* value (*n* = 7)	PTX (*n* = 57)	No-PTX	*P* value (*n* = 0)	PTX (*n* = 3)	No-PTX	*P* value (*n* = 62)
Age at diagnosis(years)	43.7 ± 12.7	49.4 ± 12.0	0.018	38.5 ± 6.9	47.4 ± 10.7	0.001	34.3 ± 12.8	43.9 ± 9.7	0.192	/	57.1 ± 10.8	NS	43.3 ± 3.2	47.6 ± 11.9	0.544
Gender															
Male	27	18	0.879	0	0	NS	4	5	1.000	/	8	NS	1	14	1.000
Female	41	29	0.879	14	27	NS	0	2	1.000	/	49	NS	2	48	1.000
BMI (kg/m^2^)	23.7 ± 2.9	23.5 ± 3.3	0.832	20.8 ± 2.6	21.1 ± 2.6	0.949	24.7 ± 3.8	23.7 ± 4.0	0.705	/	22.1 ± 3.5	NS	NA	NA	NA
Smoking status										/					
Current/former smoker	10	8	0.737	0	3	NS	4	5	1.000	/	4	NS	0	2	1.000
Never smoker	58	39	0.737	14	24	0.539	0	2	NS	/	53	NS	3	60	1.000
Family history of PTX	44	20	0.036	0	0	NS	0	0	NS	/	0	NS	0	1	NS

Definition of abbreviations: NS = non-significant; DCLDs = diffuse cystic lung diseases; BHD = Birt-Hogg-Dubé Syndrome; LAM = lymphangioleiomyomatosis; PLCH = pulmonary Langerhans cell histiocytosis; PTX = pneumothorax; SS = Sjögren’s syndrome; No-PTX = patients who did not experience pneumothorax during the study period; BMI = body mass index; / = no patients meeting the criteria; NA = not available

**Table 2:** Characteristics of pneumothorax in patients with DCLDs

Features	All patients (*n*=289)	BHD (*n*=115)	LAM (*n*=41)	PLCH (*n*=11)	SS (*n*=57)	No identifiable cause (*n*=65)
Age at first pneumothorax	33.6 yr	36.9 yr	33.1 yr	27.5 yr	NA*	37.0 yr
Average number of pneumothorax episodes	2.8	2.3	1.9	4.8	0	2.3
Number of patients with pneumothorax	89 (31%)	68 (59%)	14 (34%)	4 (36%)	0	3 (5%)
Number of patients with multiple pneumothorax	52 (18%)	41 (36%)	7 (17%)	2 (18%)	0	2 (3%)
Number of patients with bilateral pneumothorax	27 (9%)	22 (14%)	3 (7%)	2 (18%)	0	0
Recurrence rate of SP following conservative treatment	59%	69%	57%	68%	NA*	80%
Recurrence rate of SP following surgical treatment	11%	10%	20%	NA^#^	NA*	0
Follow-up duration	2.8 yr	2.2 yr	4.7 yr	3.1 yr	3.7 yr	2.1 yr

Definition of abbreviations: surgical treatment = bullectomy with or without pleurodesis, and lung transplant; conservative treatment = chest tube drainage or observation; NA = not available; DCLDs = diffuse cystic lung diseases; BHD = Birt-Hogg-Dubé Syndrome; LAM = lymphangioleiomyomatosis; PLCH = pulmonary Langerhans cell histiocytosis; SS = Sjögren’s syndrome; * = none of the patients experienced pneumothorax in SS, and related data on pneumothorax and treatment were not obtained; ^#^ = No surgical treatment was performed

## Disease subgroups

### Birt-Hogg-Dubé syndrome (BHD)

#### Baseline characteristics

A total of 115 BHD patients were identified, with average age of 46.1 ± 12.7 years, of which 61% (70/115) were female. 16% (18/115) of the patients had a history of smoking (13 current smokers, 5 former smokers), all of whom were male, and 56% (64/115) had a family history of pneumothorax. Ninety-seven (84%) of patients with BHD were diagnosed by genetic testing. Except for a 43-year-old woman diagnosed by skin biopsy revealing fibrofolliculoma, the remaining 17(15%) patients were diagnosed through familial screening. All patients met the current diagnostic criteria for BHD.

#### Pneumothorax in BHD

In the BHD cohort, with an average follow-up period of 2.2 years, 59% (68/115) of patients in this study had experienced at least one pneumothorax during their lifetime. Among the patients with pneumothorax, 156 episodes occurred in 68 patients, including 27 patients who experienced only one episode. The average age at first pneumothorax was 36.9 ± 12.7 years (range, 18–68 years), 58% of patients (67/115) were diagnosed with BHD at the time of SP including one patient who developed pneumothorax during the initial diagnostic evaluation. The average number of pneumothoraces was 2.3 (range, 1–11). Of these patients with pneumothorax, 40% had only one episode, while 60% experienced recurrences. Among those with pneumothorax, 32% (22/68) had experienced bilateral involvement, 65% (44/68) had a family history of pneumothorax, and 15% (10/68) had a history of smoking. The mean age at diagnosis of BHD was significantly lower in those with pneumothorax (43.7 ± 12.7 years) compared to those without pneumothorax (49.4 ± 12.0 years) (*P* = 0.018, 95% CI [-10.3, -1.0]).

#### Treatment and recurrence rates

Sixty-eight episodes were treated surgically; 37 had bullectomies alone, 22 had bullectomies with pleurodesis, and 9 bullectomies with unclear information about pleurodesis. Average hospital stays were 8.1 ± 2.6 days for bullectomy and 6.7 ± 3.1 days for bullectomy with pleurodesis (*P* = 0.159, 95% CI [-3.3, 0.6]). Complications in bullectomy alone cases included 2 infections and 2 prolonged air leaks, absent in other surgeries. The overall recurrence rate was 10% (7/68): 5 after bullectomy alone and 2 after bullectomy with pleurodesis. Conservative treatment (chest tube placement strategy or observation) was employed in 88 episodes with a recurrence rate of 69% (61/88). The recurrence rate after surgical management was significantly lower than that after conservative management (10% vs. 69%, *P <* 0.001, 95% CI [0.1, 0.3]).

### Lymphangioleiomyomatosis (LAM)

#### Baseline characteristics

A total of 41 patients with LAM were identified in the cohort, all of whom were female, with a mean age at diagnosis of 44.3 ± 10.4 years (range, 25–68 years). Thirty-eight (93%) patients had sporadic LAM and three (7%) had underlying tuberous sclerosis (TSC). Only three patients had a history of previous smoking, none of whom had experienced pneumothorax. Over an average follow-up period of 4.7 years, 34% (14/41) of these patients had experienced pneumothorax. The mean age at first occurrence was 33.1 years. Patients with pneumothorax were diagnosed with LAM at a significantly younger age (38.5 ± 6.9 years) than those without (47.4 ± 10.7 years) (*P* = 0.001, 95% CI [-14.50, -3.30]). The number of pneumothoraces ranged from one to five. Three patients (7%) with LAM had bilateral pneumothorax.

#### Treatment and recurrence rates

Of the 14 patients with pneumothorax, seven patients experienced only one episode, the remainder experienced multiple episodes, totaling 26 events. Among LAM patients with pneumothorax, five underwent surgery: one patient underwent lung-transplant surgery which was complicated by postoperative pneumonia resulting in a 3-month hospital stay, while the other four patients had bullectomy with pleurodesis, averaging nine days of hospitalization. Of these four patients, one patient experienced an infection, and another had pneumothorax recurrence. Thus, the overall recurrence rate of pneumothorax after surgical treatment in LAM patients was 20% (1/5). Conservatively managed patients had a 57% (12/21) recurrence rate (*P* = 0.322, 95% CI [0.1, 2.1]). Additionally, eight patients were treated with sirolimus after the initial pneumothorax (2 underwent bullectomy combined with pleurodesis and 6 received conservative management), none of them experienced recurrence of pneumothorax during follow-up (average 5.6 years).

### Pulmonary langerhans cell histiocytosis (PLCH)

#### Baseline characteristics

Of 11 patients with DCLD who had been previously diagnosed with PLCH (2 confirmed by lung biopsy, others by lymph node and bone biopsies), the mean age at diagnosis was 40.4 ± 11.7 years (range, 18–57 years), and 82% (9/11) were male. Patients with pneumothorax were diagnosed at a younger age (34.3 ± 12.8 years) compared to those without pneumothorax (43.9 ± 9.7 years), though the difference was not statistically significant (*P* = 0.192, 95% CI [-28.54, 9.34]). Of these patients, 82% (9/11) had a smoking history (7 current smokers, 2 former smokers).

#### Treatment and recurrence rates

Four patients (36%) had experienced pneumothorax; two experienced only one episode, one of whom had a history of bronchoscopy-related pneumothorax and experienced a right-sided pneumothorax during follow-up, which did not recur after closed drainage. The remaining two patients experienced recurrent pneumothoraces (10 and 7 episodes, respectively) with bilateral lung involvement. These four patients had all been managed with chest tube placement, with a total of 19 episodes of pneumothorax.

### Sjögren’s syndrome (SS)

#### Baseline characteristics

A total of 57 patients with SS-DCLD were included in this study. The mean age of patients was 57.1 ± 10.8 years (range, 35–79 years) and 49 (86%) were female. Secondary SS was diagnosed in 15 (26%) cases. Fifty-three (93%) of the patients had no history of smoking, and all four smokers were male (2 current smokers, 2 former smokers). Fifty-six (98%) patients had cystic lesions involving more than one lobe of the lung, and 55 patients (96%) had bilateral lesions. In most cases, the cysts were associated with other lesions involving the lung including bronchiectasis (*n* = 7, 12%), micronodules (*n* = 33, 57%) and air trapping (*n* = 3, 5%). The majority of patients with cystic lung disease (70%) had no respiratory symptoms. In addition to cystic lesions, 15 (26%) patients had a history of lung disease associated with SS including interstitial lung disease (ILD) (Table 3). Two patients underwent biopsies of pulmonary nodules. A 71-year-old man had a video-assisted thoracoscopic surgery (VATS) biopsy confirming lung adenocarcinoma, while a CT-guided percutaneous biopsy in a 70-year-old woman revealed benign changes.

**Table 3:** Characteristics of patients with SS-DCLD

Variable	*n* = 57
Diagnosis	
Primary SS	42 (74%)
Secondary SS	15 (26%)
Anti-SSA	56 (98%)
Anti-SSB	26 (46%)
Positive lip biopsy	35 (61%)
Symptoms	
Cough	12 (21%)
Dyspnea	17 (30%)
Sputum production	4 (7%)
Cysts on chest CT	
Number of cysts	
>10	35 (61%)
6–10	15 (26%)
3–5	7 (12%)
Size of maximum cysts	
<2 cm	25 (44%)
2–5 cm	26 (46%)
>5 cm	6 (10%)
Pulmonary complications	
Other ILD different from DCLD	15 (26%)
Multiple pulmonary nodules	33 (57%)
GGO	5 (9%)
Bronchiectasis	7 (12%)
Air trapping	3 (5%)
Pulmonary tuberculosis	2 (4%)
Pleural effusion	2 (4%)
Therapy	
Corticosteroids	49 (86%)
Immunosuppressants	48 (84%)

Definition of abbreviations: SS-DCLD = Sjögren’s syndrome-associated diffuse cystic lung disease; DCLDs = diffuse cystic lung diseases; ILD = interstitial lung disease; GGO = ground-glass opacity; Values are No. (%)

#### Pneumothorax

**Fig. 2:**
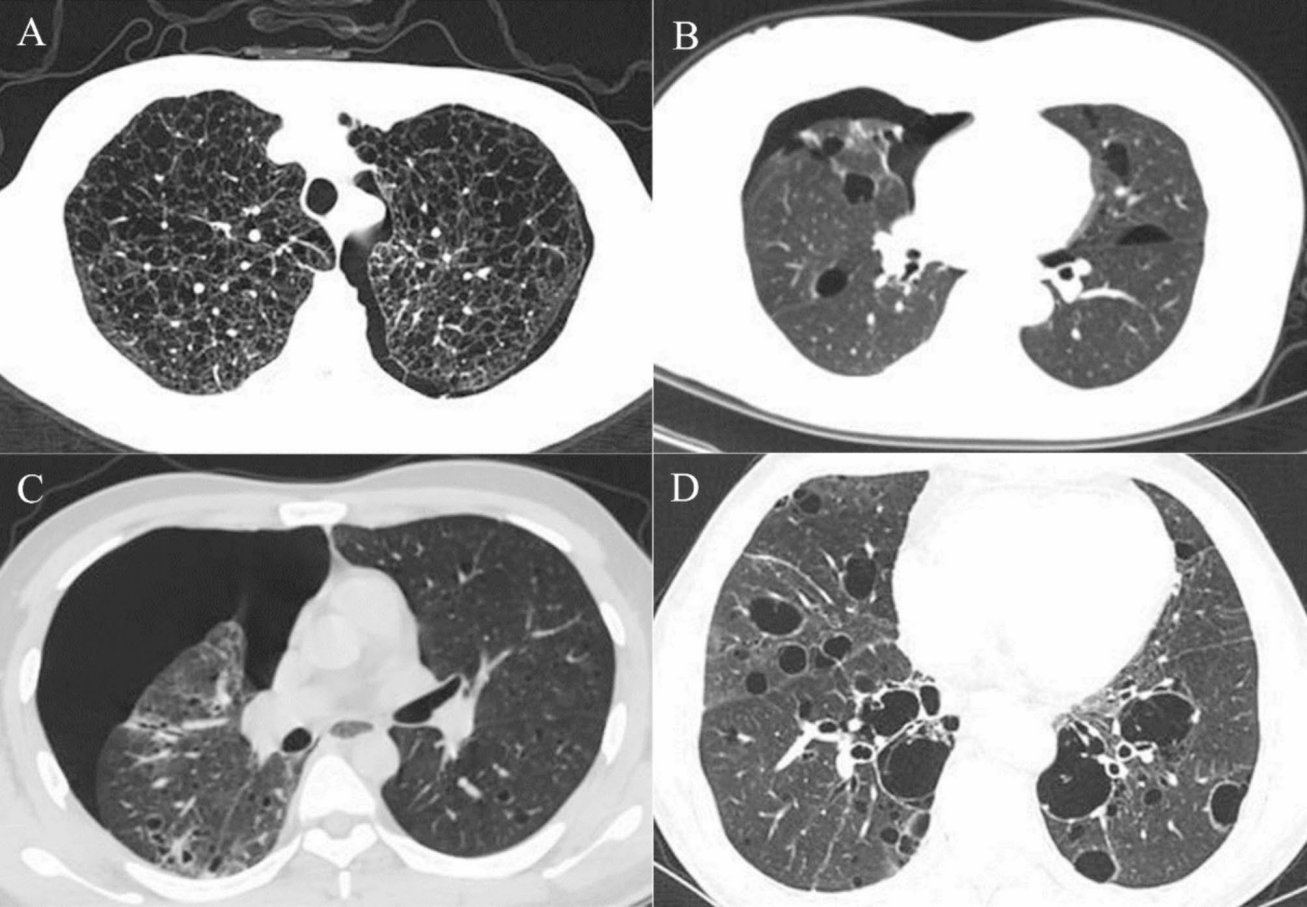
Imaging manifestations of patients with DCLDs. **A** 32-year-old female with lymphangioleiomyomatosis presenting pneumothorax. **B** 48-year-old patient with Birt-Hogg-Dubé syndrome presenting pneumothorax. **C** 30-year-old male with pulmonary Langerhans cell histiocytosis presenting pneumothorax. **D** 59-year-old elderly female with Sjögren’s syndrome demonstrated multiple cystic lung lesions

None of the SS-DCLD patients developed pneumothorax during the follow-up period of the study (Fig. 2).

### DCLD without an identifiable cause

#### Baseline characteristics

Sixty-five patients (50 females, 15 males) had no identifiable underlying disease despite extensive diagnostic testing. The mean age of patients was 47.4 ± 11.7 years (range, 25–73 years) and 50 (77%) were female. Among them, only 2 (3%) individuals were currently smoking, both male. Among DCLD patients without an identifiable cause, typical imaging features of BHD, LAM, or PLCH were absent. Of the 41 patients with complete chest CT/HRCT data, 59% had over 50 cysts, 22% had 20–50 cysts, and 19% had 3–20 cysts. All but one patient, whose cysts were confined to the right lung, showed bilateral involvement. Most cases (88%) exhibited cysts distributed throughout the lungs, while 7% showed predominant involvement of the lower lungs, and the remaining cases had upper lung involvement (Supplemental data file). Additionally, 15 patients presented multiple lung nodules or ground-glass opacities (GGOs). Laboratory testing included serum protein electrophoresis (31 patients), immunofixation electrophoresis (41 patients), anti-SSA/SSB antibodies (42 patients), and anti-neutrophil cytoplasmic antibody (5 patients), all of which were negative. Among these cases, one had renal clear cell carcinoma with unilateral cystic lesions and negative folliculin (*FLCN*) genetic testing. Two angiomyolipoma (AML) cases presented with bilateral diffuse cystic lesions atypical of LAM, with normal serum vascular endothelial growth factor-D (VEGF-D) levels.

*FLCN* genetic testing were performed on 28 patients and were negative except for one inconclusive result. Serum VEGF-D levels were measured in 42 patients, all of which were below 800 pg/ml. Lung biopsies, including one transbronchial lung biopsy (TBLB) and four via VATS were performed in five patients, none of which resulted in a definitive diagnosis.

#### Treatment and recurrence rates

Among these patients, 5% (3/65) had experienced pneumothorax, with one patient experiencing it once, and two patients experiencing multiple episodes. There were seven instances in total, with pneumothorax recurrence rates of 0% (0/2) after surgical treatment and 80% (4/5) after conservative management. Additionally, one patient reported a family history of pneumothorax, but genetic testing results were unremarkable.

## Discussion

This study draws from data spanning seven years at a Rare Disease Center in Eastern China. To our knowledge, this is the most comprehensive real-world data on DCLDs from a single center to date. In addition, it is the first study to explore the risk of pneumothorax associated with SS-DCLD. Our study reveals that SP is a relatively common feature of DCLDs, especially in patients with BHD, LAM, and PLCH, and is associated with varying prevalence and recurrence rates. Notably, SS-DCLD was found to be associated with a low risk of SP. In our study, surgical treatment of pneumothorax was associated with a significantly lower rate of recurrence overall compared to conservative management. Furthermore, sirolimus therapy may reduce the risk of SP recurrence in LAM.

SP can be the presenting manifestation of cystic lung disease [22]. Our analysis indicates that patients with DCLDs who experienced pneumothorax were diagnosed at a significantly younger age compared to those who did not. A retrospective study involving 90 PLCH cases also demonstrated that PLCH patients who experienced pneumothorax were diagnosed at a younger age compared to those who did not develop pneumothorax [16]. Additionally, these authors found recurrent pneumothorax to be diagnosed more frequently in those with delayed diagnosis of PLCH. An American survey of 104 BHD patients found 76% to have experienced pneumothorax, and in two-thirds of them, spontaneous pneumothorax was the initial manifestation that led to the diagnosis of BHD [8]. Thus, DCLDs are increasingly recognized among those with SP which serves as the initial event leading to the eventual diagnosis [23]. Previous studies have shown that BHD, LAM, PLCH, and SS [7] to be the dominant causes of DCLDs. Although pneumothorax was observed among patients with BHD, LAM, and PLCH in our study cohort, none of our patients with SS-DCLD manifested pneumothorax.

Recent studies indicate that over-activation of *TFE3/TFEB* due to *FLCN* inactivation is a key factor in the development of BHD [24, 25]. Yang et al. [26]found that the risk of pneumothorax was significantly associated with the long-axis diameter, short-axis diameter, and volume of the largest cysts in BHD. In LAM, abnormal proliferation of smooth muscle-like cells and destruction of the lung parenchyma caused by *TSC1* or *TSC2* gene mutations increase the fragility of lung tissue and the risk of pneumothorax [27]. PLCH in adults is strongly associated with smoking and is characterized by Langerhans cell infiltration. Tazi et al. [28] pointed out that in the lung tissue of PLCH patients, the accumulation of Langerhans cells causes parenchymal destruction resulting in the formation of cysts, which are prone to rupture.

SS is an autoimmune disease characterized by chronic inflammation and lymphocytic infiltration, predominantly involving T cells and B cells [29]. In SS, lung cysts are often associated with lymphoid interstitial pneumonia (LIP) [30]. These cysts can arise from ischemia resulting from vascular obstruction, post-obstructive bronchiolar ectasia, or compression of the bronchioles by lymphoid tissue, leading to subsegmental overinflation via a check-valve mechanism [31]. Steroid or immunosuppressive therapies can reduce inflammation and suppress immune responses, and are used for manifestations such as LIP [30, 32]. However, Carlos et al. observed that during a four-year follow-up period, regardless of whether the patient received immunotherapy or not, there was no radiologic progression in patients with cystic lung disease associated with SS [33]. Similarly, an observational study involving 21 patients with SS who presented with DCLD found that cystic changes are typically benign and do not substantially impact the overall prognosis associated with the disease [29]. In contrast to LAM and PLCH, cysts in SS can be randomly distributed, but often have a basilar or perivascular distribution [34]. An alternative hypothesis suggests that lymphocytic infiltration leading to the destruction of alveolar walls as the underlying cause of cyst formation [35, 36].

CT imaging can be a helpful tool for the early detection of cystic lung diseases in patients who experienced spontaneous pneumothorax [37]. The distinct distribution pattern and distribution of cysts in DLCDs aid clinicians in suspecting the underlying disease. HRCT of the chest in patients with LAM typically reveals diffuse thin-walled, round cysts with no other findings in the intervening lung parenchyma [38]. Characteristic HRCT features of BHD include thin-walled, round to oval-shaped cysts of varying sizes, mainly in the lower and subpleural lung areas [39]. HRCT is crucial for diagnosing PLCH, with findings primarily concentrated in the upper and middle lung zones, typically showing a combination of nodules including those with cavitation and cysts [40]. Given the rarity of these diagnoses and the costs and radiation involved, routine chest CT for all SP patients is not recommended. Instead, a focused history and physical examination should guide the decision, emphasizing recurrent pneumothorax, family history, and signs of underlying diseases like BHD or TSC. If findings suggest a potential DCLD, CT imaging can be pursued.

In addition, family history of pneumothorax can be a significant indicator in diagnosing BHD [41, 42]. Liu et al. performed *FLCN* genetic testing on 25 probands with familial spontaneous pneumothorax, and pathogenic mutations were identified in 64% of them [15]. In another study, *FLCN* genetic testing was conducted on 7 patients with SP who also had a family history of pneumothorax. Of these, 6 out of 7 patients tested positive for *FLCN* gene mutations, and all patients with positive *FLCN* genetic testing were found to have lung cysts [43]. Hu et al. reported that up to 85% of BHD patients had a family history of pneumothorax in China [44]. Additionally, several studies have reported the correlation between genetic phenotypes and pneumothorax [42, 45]. In the future, large cohort studies are needed to further explore the relationship between genotype and phenotypic expression in BHD. A recent study in Japan identified BHD as a significant risk factor for pneumothorax, with an occurrence rate as high as 72%, compared to 7.8% in Marfan syndrome and 7.5% in Ehlers-Danlos syndrome among heritable connective tissue disorders [46]. Similarly, our study found a high prevalence of pneumothorax in BHD patients (59%), 65% of whom had a familial history of the condition. In contrast, patients with LAM (including sporadic LAM and TSC-LAM), PLCH, and SS did not demonstrate this familial feature in our study cohort. Therefore, when DCLDs patients with presenting positive family history of pneumothorax, BHD should receive the highest consideration, including genetic testing.

In current study, the recurrence rate of pneumothorax was higher with observation or chest tube placement strategy compared to surgical treatment. A retrospective study from the Peking University People’s Hospital demonstrated that the recurrence rate of pneumothorax in BHD patients was only 9.1% after surgical treatment, compared to 53.1% with conservative treatment [15]. Mendez and colleagues [47] reported a high pneumothorax recurrence rate of 58% following conservative treatment with chest tube drainage, whereas there was no recurrence observed after thoracotomy in PLCH. A study using a nationwide Japanese inpatient database spanning nearly a decade found that surgical treatment for secondary spontaneous pneumothorax (SSP) in patients with heritable connective tissue disorders had a notably lower recurrence rate (26%) compared to non-surgical treatment (44%) [46]. Similarly, in a survey of 193 LAM patients with pneumothorax, the recurrence rates were 66% after conservative treatment, 27% after chemical pleurodesis, and 32% after surgery [14]. Overall, the reported recurrence rates for pneumothorax after surgical treatment is significantly lower than after conservative treatment. The latest guidelines for the management of SP [48] noted that there was no significant difference in outcomes between SP patients who underwent only lung surgery compared to those who had both lung surgery and pleurodesis, and suggested lung surgery alone can effectively prevent pneumothorax recurrence. However, due to potential bias of retrospective case series, a multicenter randomized controlled trial directly comparing surgical pleurectomy and chemical pleurodesis is warranted to clarify this issue.

In LAM, sirolimus therapy can prevent disease progression by inhibiting the mTOR pathway. Several studies reported sirolimus therapy may reduce the recurrence of pneumothorax [49–51]. None of the eight LAM patients with pneumothorax in our cohort had recurrence during the follow-up period after treatment with sirolimus, which is in line with the results of previous studies. However, given the small number of patients, further studies are needed to confirm the potential impact of sirolimus therapy on reducing recurrence of pneumothorax in patients with LAM.

Our study has several limitations. Firstly, the reliance on recall for eliciting some of the pneumothorax data posed a potential limitation, especially 9 (10%) older patients who may have struggled to accurately remember their episodes of pneumothorax and the details of their surgical treatments. Additionally, we were unable to determine the exact interval between the initial pneumothorax episode and chest CT scan being performed. Secondly, inconsistent use of genetic testing and lung biopsy for patients with undiagnosed DCLDs presents another challenge. Finally, as the diagnosis of LIP/amyloidosis necessitates a lung biopsy, we cannot definitively establish the underlying pathology for all cases of SS-DCLD. Furthermore, previous literature has reported a rare case of SS coexisting with BHD [52]. Due to cost and patient availability issues, genetic testing was not conducted on all SS patients to exclude the possibility of concurrent BHD.

## Conclusion

In this study, the highest risk of SP was observed in DCLDs associated with BHD, followed by those with PLCH and LAM. Conversely, the risk of pneumothorax in patients with SS-DCLD was notably low. Pneumothorax can be the initial manifestation of DCLDs, often facilitating diagnosis of underlying disorders. Surgical intervention significantly lowered the recurrence rate of pneumothorax among patients with DCLDs. Additionally, sirolimus therapy may reduce the risk of pneumothorax recurrence in patients with LAM.

## Electronic supplementary material

Below is the link to the electronic supplementary material.

Supplementary Material 1

## Data Availability

The datasets generated and analyzed for this study are not publicly available due to participant privacy but are available from the corresponding author upon reasonable request.

## Declarations

### Statement

The abstract of this article has been accepted for presentation at the CHEST 2024 Annual Meeting. Currently, the relevant DOI and conference paper link have not been assigned.

## Abbreviations

AML: Angiomyolipoma
BHD: Birt-Hogg-Dubé syndrome
CT: Computed tomography
DCLDs: Diffuse cystic lung diseases
DCLD: Diffuse cystic lung disease
FLCN: Folliculin
HRCT: High-resolution CT
ILD: Interstitial lung disease
LAM: Lymphangioleiomyomatosis
LIP: Lymphoid interstitial pneumonia
PLCH: Pulmonary Langerhans cell histiocytosis
SP: Spontaneous pneumothorax
SSP: Secondary spontaneous pneumothorax
SS: Sjögren’s syndrome
SS-DCLD: Sjögren’s syndrome-associated diffuse cystic lung disease
TSC: Tuberous sclerosis
TBLB: Transbronchial lung biopsy
USTC: University of Science and Technology of China
VATS: Video-assisted thoracoscopic surgery
VEGF-D: Vascular endothelial growth factor-D
